# *In Vivo* Initiation of Clock Gene Expression Rhythmicity in Fetal Rat Suprachiasmatic Nuclei

**DOI:** 10.1371/journal.pone.0107360

**Published:** 2014-09-25

**Authors:** Pavel Houdek, Alena Sumová

**Affiliations:** Department of Neurohumoral Regulations, Institute of Physiology, Academy of Sciences of the Czech Republic, Prague, Czech Republic; University of Lübeck, Germany

## Abstract

The mammalian suprachiasmatic nuclei (SCN) and their intrinsic rhythmicity develop gradually during ontogenesis. In the rat, the SCN forms between embryonic day (E) 14 and E17, with gestation terminating at E21–22. Overt SCN rhythmicity is already present in the late embryonic stage. The aim of the present study was to determine when the fetal SCN clock develops in vivo and whether overt rhythmicity results from a functional fetal clock. To achieve this goal, the prenatal development of rhythmic expression of clock genes was measured with a more sensitive method for detection of the clock gene expression than previously. Fetal SCN were collected at 3 h intervals during the 24 h period on E19 and E21 by laser dissection and expression of clock genes (*Per2*, *Nr1d1* and *Bmal1*) and genes related to cellular activity (*c-fos*, *Avp* and *Vip*) was measured by qRT PCR. At E19, the expression of canonical clock genes *Per2* and *Bmal1* was not rhythmic; however, the expression of all other studied genes followed clear circadian rhythms. At E21, *Per2* and *Bmal1* expression exhibited low amplitude but significant rhythmicity. From E19 to E21, the levels of the non-rhythmic transcripts (*Per2* and *Bmal1*) decreased; however, the levels of the rhythmic transcripts (*Nr1d1*, *c-fos*, *Avp* and *Vip*) increased. In summary, these data demonstrate that at E19, rhythms in *Per2* and *Bmal1* expression were absent in the fetal SCN; however, the expression of *Nr1d1* and other genes related to cellular activity was driven rhythmically. Therefore, at the early stage in vivo, the developing fetal SCN clock could theoretically be entrained by oscillation of *Nr1d1* which may be driven by the maternal rather than fetal circadian system.

## Introduction

In mammals, the suprachiasmatic nuclei of the hypothalamus (SCN) are a site of the principal pacemaker [1], which drives overt behavioral, physiological, hormonal, biochemical and molecular rhythms (for review, see [2]). These rhythms repeat with a period of approximately 24 h and are thus termed “circadian”. Overt circadian rhythms can be detected in the SCN themselves, namely in metabolic [3] and electrical [4] activity. At the molecular level, overt circadian rhythms manifest in spontaneous expressions of genes related to neuronal activity, e.g., immediate early gene *c-fos* and *arginine vasopressin* (*Av*p) [5]–[7]. In adults, the SCN drives these overt rhythms via a cell-autonomous molecular clockwork (for review, see [8]). This clock mechanism is based on the rhythmic expression of so-called clock genes. The rhythm is endogenously generated and maintained due to the mechanism in which the clock gene protein products feed back onto promoters of the clock genes and activate or inhibit their transcription. Clock proteins CLOCK an BMAL1 serve as transcriptional activators that switch on transcription of clock genes *Per1,2*, *Cry1,2* and *Nr1d1* (*Rev-erbα*). PER1,2 and CRY1,2 proteins serve as inhibitors of the CLOCK:BMAL1 activated transcription and NR1D1 directly inhibits *Bmal1* expression. As a result, in the SCN, the expression of *Per1,2*, *Cry1,2*, *Nr1d1* peaks during the subjective day, whereas that of *Bmal1* peaks during the subjective night. Importantly, the presence of circadian variation in transcript levels is conditional for clock “ticking”. The expression of *Per1* and *Per*2, but not of other clock genes, is activated by light at night [9], [10], which represents a mechanism for entrainment of the molecular clock by the light/dark cycle. The SCN is a multi-cellular clock whose properties are dependent on a synaptic web ensuring communication among individually rhythmic cells. This unique feature of the SCN provides the central clock with its robustness, plasticity and ability to adjust to changes in external environment [11], [12]. The molecular mechanism is also present in other neuronal and non-neuronal cells of the body, i.e., so-called peripheral oscillators. Importantly, the peripheral clocks are not directly synchronized by light but via by not yet fully recognized local signals and systemic signals derived from the SCN [13]. Systemic signals set the phases of the peripheral oscillators relatively to the SCN by modulating the expression of some of the clock genes of the peripheral clocks [14].

During ontogenesis, SCN morphology and rhythmicity develop gradually (for review, see [15]). In the rat, gestation takes 22 days and the nuclei are formed from the embryonic day (E) 14 through E17 from the specialized zone of the ventral diencephalic germinal epithelium as a component of the periventricular cell groups [16]. Whereas neurogenesis is completed at approximately E17, SCN morphology is not yet complete because the individual SCN neurons are not mutually interconnected by synapses. Synaptogenesis progresses slowly during the late prenatal stage between E19 and parturition, when only few synapses appear in the SCN; however, their number increases rapidly postnatally, with a marked rise from postnatal day (P)4 to P10 [16].

Despite its immaturity, intrinsic rhythmicity is already present in the SCN structure in late stages of embryogenesis. Clear day-night oscillation in metabolic activity was detected in the fetal rat SCN already from E19 through E21 by monitoring 2-deoxyglucose uptake [17]. However, rhythmicity in *Avp* expression is not present before E20 [18], [19], and rhythmic firing rates in SCN neurons appear only at E22 [20]. To determine if these rhythms are driven by the fetal SCN clock, daily profiles of clock gene expression were measured during the prenatal period by in situ hybridization. At E19, an early stage in rat SCN development, no significant circadian rhythmicity was detected in *Per1*, *Per2*, *Cry1*, *Bmal1* expression [21]. One day later, at E20, rhythmic expression of *Per1* and *Per2* was reported [22], [23]. The robustness of clock oscillation further develops postnatally, in correlation with synaptogenesis; in the rat, the amplitude of clock gene expression rhythms increased up to P5–P10 [19], [21]. In mice, the gestation takes 18 days and a daily rhythm of *Per1*, but not of *Per2*, expression was detected at E17 in the SCN. The rhythm in *Per2* expression was not present at P3 and it appeared only at P6 [24]. The aim of the present study was to test the hypothesis that at the early fetal SCN development, in vivo oscillation is driven by maternal rhythmic cues before an autonomous clock mechanism is established. To test this hypothesis, we compared the daily profiles of clock gene transcript levels (*Per2*, *Nr1d1* and *Bmal1*) in the fetal SCN at two developmental stages, namely at E19 and E21. Additionally, daily expression profiles of *c-fos*, a marker of neuronal activity, and *Avp* and *Vip*, two main SCN neurotransmitters, were measured. In contrast to previous studies, which used in situ hybridization to detect the transcript levels, in this study we used a more sensitive quantitative RT-PCR method. In the fetal SCN, most of the transcript levels were found to be relatively low and, therefore, there was a possibility that their circadian variation could be missed especially in case when the background staining of the in situ hybridization probe was higher. Using the quantitative RT-PCR overcomes this methodological difficulty and provides more precise detection of daily variations in the transcript levels. The results revealed that in vivo, circadian rhythms in gene expression are present in the fetal SCN before the functional molecular clock develops.

## Materials and Methods

### Ethics statement

All experiments were approved by the Animal Care and Use Committee of the Institute of Physiology and were in agreement with the Animal Protection Law of the Czech Republic, as well as the European Community Council directives 86/609/EEC. All efforts were made to ameliorate the suffering of the animals.

### Experimental animals

Adult Wistar rats (Bio Test s.r.o., Konarovice, Czech Republic) were housed in a temperature-controlled facility at 23±2°C with free access to food and water. Animals were maintained under a light/dark cycle with 12 h of light and 12 h of darkness (LD12∶12) for at least 2 months. Light was provided by overhead 40-W fluorescent tubes, and illumination was between 50 and 200 lx, depending on cage position in the animal room. Female rats were inspected by vaginal smears every day to determine estrous cycle phase. On the night of proestrus, they were mated with males; day 0 of embryonic development (E0) was defined as the day vaginal smears were found to be sperm-positive. Lights-on time was designated Zeitgeber time 0 (ZT0), and lights-off time as ZT12. Mothers were sacrificed in 3 h intervals throughout the entire 24 h cycle. For profiles at E19, the sampling was performed from ZT12 on E18 until ZT12 on E19, and for the profiles at E21, the sampling started at ZT12 on E20 and was terminated at ZT12 on E21. At each time point, one pregnant rat was sacrificed and 6–8 (occasionally 10) fetuses were sampled. Fetuses were sacrificed by rapid decapitation, and the heads were immediately frozen in dry ice and stored until sectioning and SCN separation.

### Laser capture microdissection

**Figure 1:**
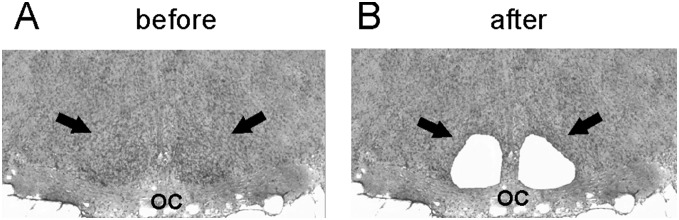
Representative coronal sections of the fetal rat brain at embryonic day 21 containing the hypothalamus. The sections were stained with cresyl violet and the position of the suprachiasmatic nuclei (SCN) is denoted (arrows). **A**. The intact section before the SCN dissection; **B**. The same section after the SCN dissection. For more details, see Materials and Methods. OC, optic chiasma.

Brains were sectioned on cryocut into 20 µm thick sections of hypothalamus containing the medial part of the rostro-caudal extent of the SCN. The sections were stained with cresyl violet (Sigma Aldrich, St. Louis, USA). The SCN was then precisely separated bilaterally from the remainder of the section using a laser microdissector (LMD6000, Leica). Representative sections of the hypothalamic area before and after SCN laser dissection are depicted in Fig. 1. Dissected samples from each section were collected in a microfuge tube containing RLT buffer from the RNeasy Micro kit (Qiagen, Valencia, USA) and stored until RNA isolation.

### RNA isolation and real-time RT-PCR

Total RNA was isolated using the RNeasy Micro kit (Qiagen, Valencia, USA) according to the manufacturer’s instructions. Isolated RNA samples were immediately reverse-transcribed into cDNA using the SuperScript VILO cDNA Synthesis Kit (Invitrogen, Carlsbad, USA). The cDNA samples were analyzed by real-time PCR on a ViiA7 Real-Time PCR System (Life Technologies, Carlsbad, USA) using 5x HOT FIREPol Probe qPCR Mix Plus (Solis Biodyne, Tartu, Estonia) and TaqMan Gene Expression Assays (Life Technologies) specific for rat gene *Bmal1* (cat. no. Rn00577590_m1), *Per2* (cat. no. Rn01427704_m1), *Nr1d1* (cat. no. Rn01460662_m1), *Avp* (cat. no. Rn00566449_m1), *Vip* (cat. no. Rn01430567_m1) and *c-fos* (cat. no. Rn02396759_m1). The assay probes span exon junctions. Expression of the housekeeping gene glyceraldehyde-3-phosphate dehydrogenase (GAPDH, Rat GAPDH Endogenous Control cat. No. 4352338, Applied Biosystems, Foster City, USA) was measured in a duplex reaction to normalize the mRNA concentrations. A single PCR reaction was performed in a final volume of 20 µl; target gene probes and the GAPDH probe were dye-labeled with FAM (6-carboxyfluorescein) and VIC (4,7,2′-trichloro-7′-phenyl-6-carboxyfluorescein) fluorescent dyes, respectively. The ΔΔCt method was used for the quantification of relative cDNA concentration.

### Statistical analysis

Gene expression profiles were analyzed for rhythmic and non-rhythmic expression by fitting to two alternative regression models: data were fitted to either a horizontal straight line (null hypothesis) or a single cosine curve (alternative hypothesis) defined by the equation Y = mesor+(amplitude*cos(2*π*(X-acrophase)/wavelength)) with a constant wavelength of 24 h. The extra sum-of-squares F test was used for comparison, and when the P value exceeded 0.05, cosine curve parameters were calculated. The amplitude (the difference between the peak or trough and the mean value of a cosine curve), acrophase (the phase angle of the peak of a cosine curve), mesor (the average value around which the variable oscillates) and coefficient of determination R^2^ (goodness of fit) were evaluated. The least-squares regression method implemented by the Prism 5 software (GraphPad, La Jolla, USA) was applied.

The levels of each individual transcript at the ZTs when maximal and minimal levels occurred were compared at E19 and E21 by one-way ANOVA with the Student-Newman-Keuls multiple comparison method; P<0.05 was required for significance.

## Results

### Daily profiles of gene expression in the fetal SCN at E19

**Figure 2:**
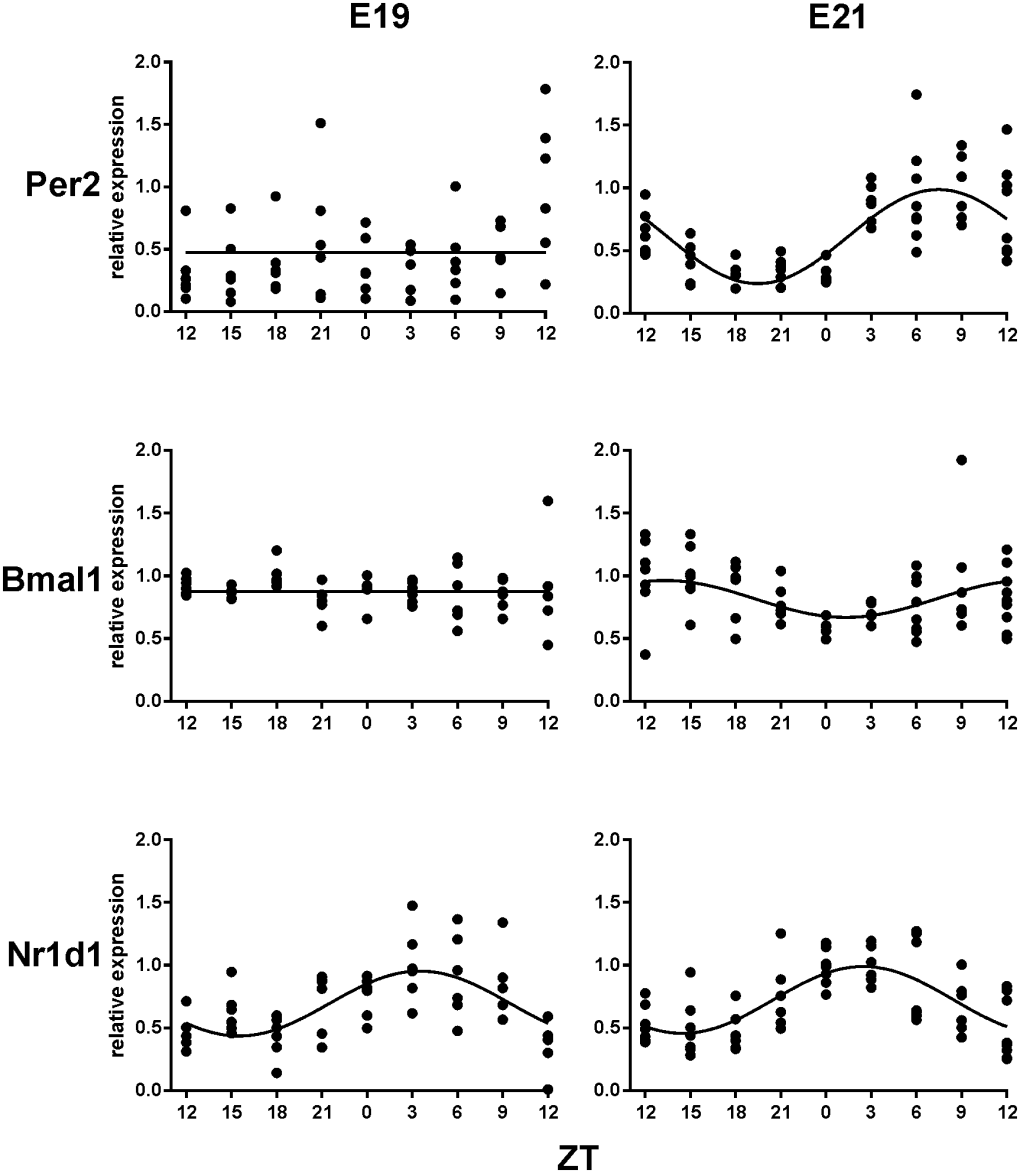
Clock gene expression profiles in the fetal rat SCN. 24-h profiles of clock gene (*Per2*, *Bmal1* and *Nr1d1*) expression were detected at embryonic day 19 (E19) (left column) and embryonic day 21 (E21) (right column). Relative expression levels of the clock genes in the individual SCN are shown. The data were analyzed with cosinor analysis and the presence of significant circadian variation in the transcript levels is denoted by a cosine curve whereas its absence is denoted by a straight line. For details, see Materials and Methods. Time is presented as Zeitgeber time (ZT); ZT0 corresponds to the time of lights-on and ZT12 to lights-off.

The daily expression profiles of clock genes *Per2*, *Nr1d1* and *Bmal1* in the fetal SCN at E19 are depicted in Fig. 2 (left column). Results of cosinor analysis of the gene expression profiles are summarized in Table 1. Cosine fit analysis indicated that *Per2* and *Bmal1* expression did not exhibit significant circadian variations. In contrast, *Nr1d1* expression exhibited a circadian rhythm with an acrophase at ZT 3.5±0.7 h.

**Table 1:** Cosinor analysis of expression profiles of clock gene *Per2*, *Nr1d1* and *Bmal1* in the SCN of fetuses at E19 and E21.

	Cosinor data	*Per2*	*Nr1d1*	*Bmal1*
	**Acro ± SEM**	-	3.5±0.7	-
**E19**	**Amp ± SEM**	-	0.588±0.111	-
	**R^2^**	0.025	0.362	0.010
	**P**	0.5274	**<0.0001**	0.7688
	**Acro ± SEM**	7.5±0.4	2.5±0.6	13.3±1.3
**E21**	**Amp ± SEM**	0.607±0.077	0.520±0.076	0.158±0.051
	**R^2^**	0.518	0.445	0.147
	**P**	**<0.0001**	**<0.0001**	**0.0116**

Acro (acrophase); Amp (amplitude); R^2^ (coefficient of determination); 19-day old fetuses (E19) and 21-day-old fetuses (E21).

**Figure 3:**
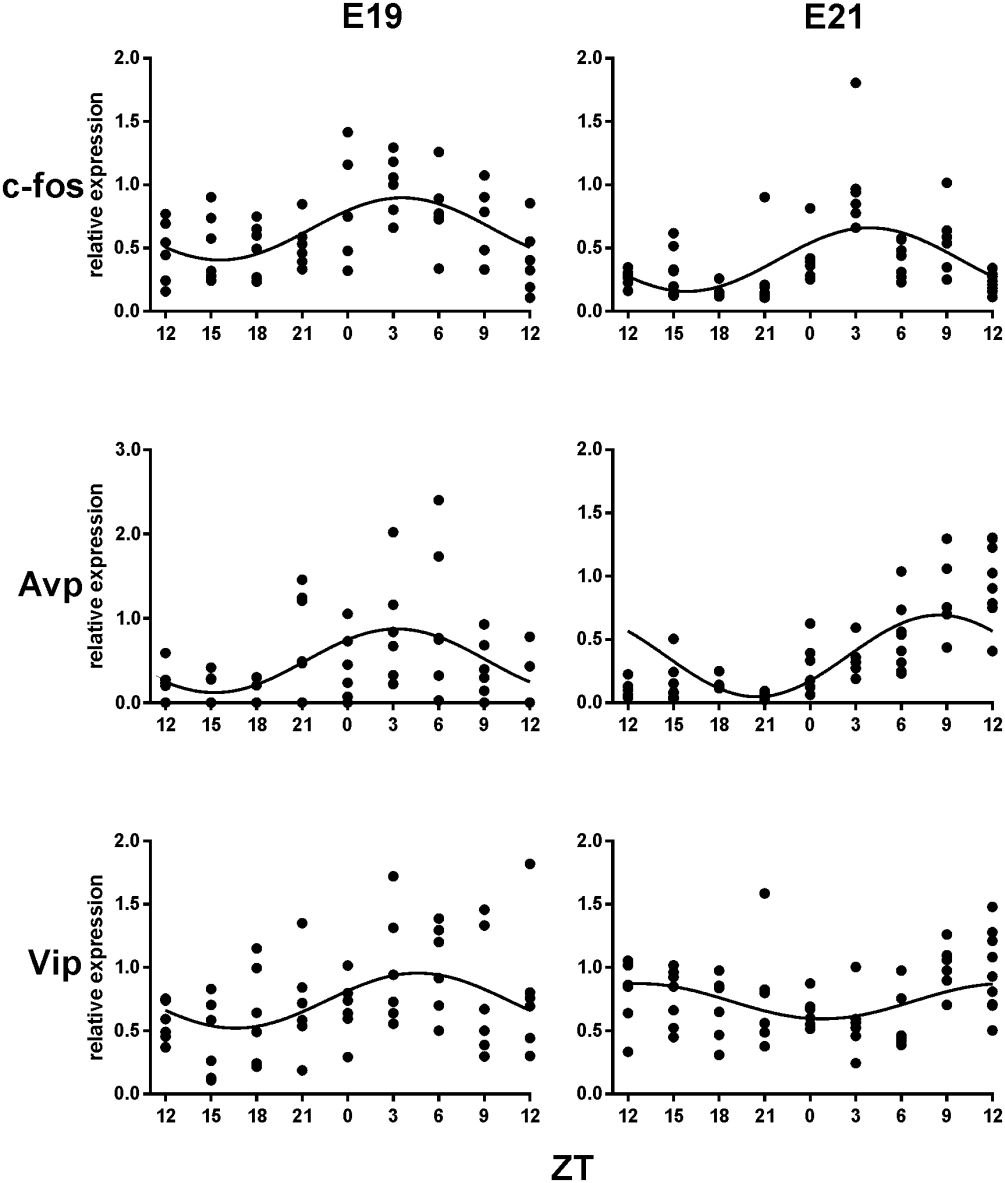
The gene expression profiles in the fetal rat SCN. The 24-h profiles of *c-fos*, *Avp* and *Vip* were detected at E19 (left column) and E21 (right column). For further details, see legend to Figure 2.

The daily expression profiles of *c-fos*, *Avp* and *Vip* in the fetal SCN at E19 are depicted in Fig. 3 (left column). The results of cosinor analysis are summarized in Table 2. All three genes were expressed rhythmically. *c-fos* and *Avp* expression profiles exhibited significant circadian rhythms with the acrophases at ZT 3.6±0.8 h and ZT 3.2±1.0 h, respectively. *Vip* expression exhibited a rhythm with lower amplitude and a peak at ZT 4.6±1.2 h.

**Table 2:** Cosinor analysis of *c-fos*, *Avp* and *Vip* gene expression profiles in the SCN of fetuses at E19 and E21.

	Cosinor data	*c-fos*	*Avp*	*Vip*
	**Acro ± SEM**	3.6±0.8	3.2±1.0	4.6±1.2
**E19**	**Amp ± SEM**	0.596±0.131	0.559±0.143	0.397±0.138
	**R^2^**	0.292	0.235	0.140
	**P**	**0.0001**	**0.0012**	**0.0213**
	**Acro ± SEM**	3.9±0.7	8.5±0.7	12.8±1.5
**E21**	**Amp ± SEM**	1.041±0.197	3.280±0.628	0.192±0.066
	**R^2^**	0.327	0.327	0.127
	**P**	**<0.0001**	**<0.0001**	**0.0197**

Acro (acrophase); Amp (amplitude); R^2^ (coefficient of determination); 19-day old fetuses (E19) and 21-day-old fetuses (E21).

In summary, expression of clock genes *Per2* and *Bmal1*, which are the core elements of the molecular circadian clock, was not rhythmic at E19. The rhythmic expression profiles of *Nr1d1, c-fos*, *Avp* and *Vip* peaked at about the same time and thus they were apparently all in the same phase.

### Daily profiles of gene expression in the fetal SCN at E21

Daily expression profiles of the clock genes *Per2*, *Nr1d1* and *Bmal1* in the fetal SCN at E21 are depicted in Fig. 2 (right column). The results of cosinor analysis are summarized in Table 1. In contrast to E19, all clock genes were expressed rhythmically. However, the rhythmicity of *Bmal1* expression, which peaked at ZT 13.3±1.3 h, was still very weak; its amplitude and R^2^ value were much lower than that observed in other rhythmic genes. The daily profiles of *Per2* and *Nr1d1* expression exhibited significant circadian rhythms and peaked at 7.5±0.4 and 2.5±0.6, respectively. Therefore, at E21, the weak rhythmicity in *Bmal1* expression was roughly in antiphase with *Nr1d1*, and the rhythm of *Per2* expression was delayed to *Nr1d1*. This proper phasing of clock gene expression suggests that at E21, the circadian clock in the SCN is functional.

Daily expression profiles of *c-fos*, *Avp* and *Vip* in the fetal SCN at E21 are depicted in Fig. 3 (right column). The results of cosinor analysis are summarized in Table 2. Similar to E19, all three genes were expressed rhythmically at E21. Expression of *c-fos* and *Avp* exhibited robust rhythms, whereas that of *Vip* was very weak. The acrophase of the *c-fos* rhythm was at ZT 3.9±0.7 h, similar to that at E19. The *Avp* and *Vip* rhythms shifted relative to E19; their acrophases were at ZT 8.5±0.7 h and ZT 12.8±1.5 h, respectively. Additionally, the amplitude of the *Avp* expression rhythm increased significantly relative to E19.

Altogether, these data suggest that a functional circadian clock operates in vivo at E21 and initiates driving the clock-controlled gene expression.

### Comparison of gene expression levels between E19 and E21

**Figure 4:**
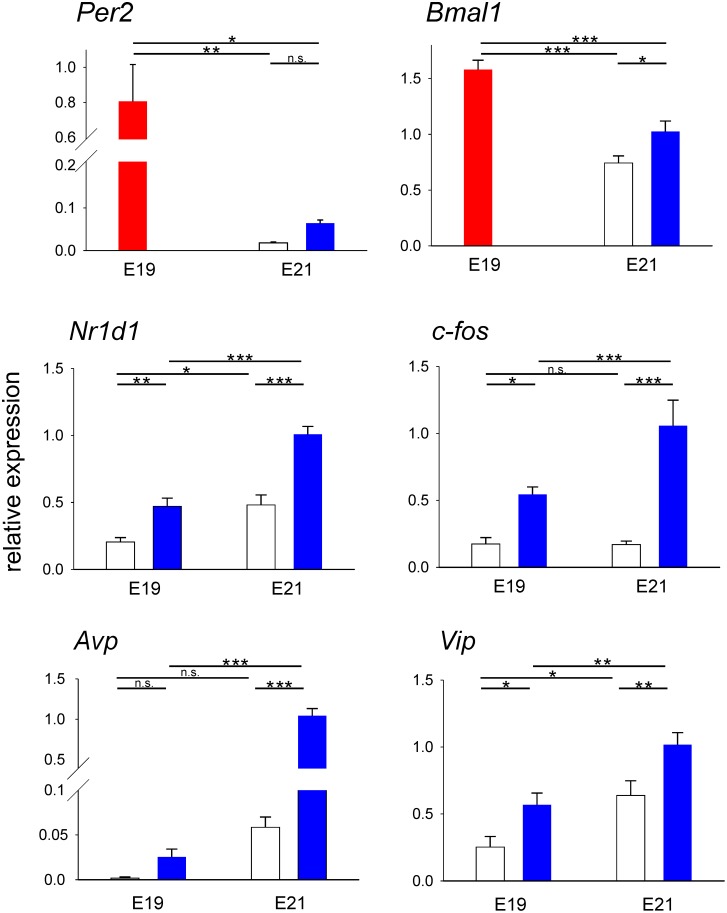
The comparison of gene expression levels in the rat SCN at E19 and E21. Relative expression of *Per2*, *Bmal1*, *Nr1d1*, *c-fos*, *Avp* and *Vip* was detected at both fetal stages. SCN samples collected at time points representative of minimal (open columns) and maximal (blue columns) transcript levels during the 24-h interval (see Fig. 2 and Fig. 3) were assayed in the same PCR assay. For the non-rhythmically expressed *Per2* and *Bmal1* at E19, the mean of maximal and minimal levels is shown (red column). The results of a one-way ANOVA comparison between the groups are shown. *P<0.05, **P<0.01, ***P<0.001, n.s., non-significant.

To compare expression levels of *Per2*, *Nr1d1*, *Bmal1*, *c-fos*, *Avp* and *Vip* in the SCN at E19 and E21, selected samples from each profile at both fetal stages were assayed in the same PCR. From each daily rhythmic profile, samples were selected from two ZTs, corresponding to the maximal and minimal expression levels during the 24 h. For non-rhythmic profiles (*Per2* and *Bmal1* at E19), expression was calculated as the mean from samples collected at ZTs when the highest and lowest expression levels were detected. The results are depicted in Fig. 4. For each of the studied genes, analysis by one-way ANOVA revealed a significant effect of the groups (*Per2*: F = 6.993, P = 0.005; *Bmal1*: F = 30.555, P<0.001; *Nr1d1*: F = 31.080, P<0.001; *c-fos*: F = 18.432, P<0.001; *Avp*: F = 99.621, P<0.001; *Vip*: F = 15.133, P<0.001). The results of the post hoc analyses are as follows:

For *Per2* (Fig. 4A), the mean expression level of the non-rhythmic profile at E19 was significantly higher than the maximal (ZT9) and minimal (ZT18) expression levels at E21 (P = 0.012 and P = 0.010, respectively). However, there was no significant difference between the minimal and maximal levels at E21, despite the significant circadian variation indicated by cosinor analysis.

Similarly for *Bmal1* (Fig. 4B), the mean expression of the non-rhythmic profile at E19 was also significantly higher than the maximal (ZT12) and minimal (ZT0) expression levels at E21 (both P<0.001). At E21, the minimal levels were significantly lower than the maximal levels (P = 0.033), coincident with detection of a significant circadian rhythm by cosinor analysis.

For *Nr1d1* (Fig. 4C), which was rhythmically expressed at both fetal stages, the minimal and maximal expression levels differed significantly at both E19 (ZT18 vs. ZT6; P = 0.008) and E21 (ZT12 vs. ZT3; P<0.001). Overall *Nr1d1* expression increased with fetal age, evidenced by increases in both the minimal and maximal expression levels between E19 and E21 (P = 0.017 and P<0.001, respectively).

For *c-fos* (Fig. 4D), the minimal and maximal expression levels were significantly different at E19 (ZT12 vs. ZT3; P = 0.018) and E21 (ZT18 vs. ZT3; P<0.001). The maximal (P<0.001) but not minimal expression increased between E19 and E21.

For *Avp* (Fig. 4E), the minimal and maximal levels at ZT15 and ZT3, respectively, did not differ significantly at E19, although the cosinor analysis detected a significant circadian variation at the fetal stage. At E21, the minimal and maximal levels already differed significantly (ZT21 vs. ZT9; P<0.001). The maximal expression increased significantly between E19 and E21 (P<0.001).

For *Vip* (Fig. 4F), the minimal and maximal levels differed significantly at both E19 (ZT15 vs. ZT6; P = 0.012) and E21 (ZT0 vs. ZT12; P = 0.005). Overall *Vip* expression increased significantly with the fetal age, evidenced by the significant rise in both minimal (P = 0.012) and maximal (P = 0.003) expression levels.

These results show that constitutively expressed genes at E19 (*Per2* and *Bmal1*) were expressed at high levels, and their expression declined with fetal age. In contrast, expression of genes expressed rhythmically at E19 (*Nr1d1*, *c-fos*, *Avp* and *Vip*) increased with fetal age.

## Discussion

Our results revealed a gradual development of circadian oscillations in individual clock gene expression in the SCN during late fetal development in vivo. These data suggest that the fetal clock does not operate at E19 but is functional at E21, immediately before parturition. In this study, transcript levels were quantified in laser-dissected SCN tissue by RT PCR, which allowed us to study the developing rhythms in more detail and with higher resolution. Hence, weak rhythms or rhythms of low-abundance transcripts could be revealed. These results are in agreement with our previous data, in which the transcript levels in the fetal SCN were detected using in situ hybridization [19], [21], a less sensitive method.

Whereas the molecular core clock components *Per2* and *Bmal1* were not expressed rhythmically at E19, expression became rhythmic at E21. In contrast, the clock gene *Nr1d1* was rhythmically expressed at both E19 and E21. The presence of the rhythm confirms that the lack of rhythmicity in *Per2* and *Bmal1* expression at E19 did not occur because the profiles of individual fetuses were rhythmic but not mutually synchronized. *Per2* encodes the PER2 protein, which is negative element of the transcriptional-translational feedback loop (for review, see [8]); the rhythm of its availability is necessary for the rhythmic suppression of CLOCK:BMAL1- activated E-box mediated gene transcription. The absence of rhythmic expression in *Per2* and *Bmal1* suggests that the expression of other clock genes is neither repressed nor activated rhythmically at E19; thus, the clock likely does not operate autonomously. This hypothesis is supported by the results of our previous study; we observed constitutive expression of not only *Per2* and *Bmal1* but also of other canonical clock genes, namely *Per1* and *Cry1*, in the SCN at E19 [21]. NR1D1 represents a core clock component of the molecular clock which is also rhythmically activated by CLOCK:BMAL1 via an E-box on its promoter. NR1D1 suppresses *Bmal1* expression during the subjective day [25]. The rhythmicity of its expression may be driven not only by the clock mechanism, but also by other pathways because its promoter region contains various response elements [26], [27]. NR1D1 represents a formally classified orphan receptor whose ligands (heme, carbon monoxide) have been identified [28], [29] and thus, daily oscillations in these ligands, driven by maternal metabolic cues, might rhythmically activate NR1D1. Because of an auto-feedback loop in which NR1D1 binds its own promoter [30], the imposed rhythm in NR1D1 activity may drive rhythm in *Nr1d1* expression, independent of fetal clock silence. In support of this scenario, *Nr1d1* rhythmicity independent of rhythm in other canonical clock genes has been observed in the intestine clocks of behaviorally arrhythmic adults rats maintained in LL [31].

Therefore, our results favor the hypothesis that the clock does not operate in the SCN at E19 in vivo; however some genes are expressed rhythmically due to rhythmic maternal drive [15]. Indeed, rhythmicity in expression of genes related to cellular activity, namely *c-fos* and *Avp*, was present already at E19. Interestingly, transcription of these two genes is activated by similar signaling pathways; both are regulated by phosphorylation of CREB (cAMP response element binding protein) because their promoters contain a responsive element (CRE) which binds P-CREB [32], [33]. This pathway represents a common signal responding to various stimuli. Therefore, these stimuli may be derived from maternal rhythmic cues, which elevate P-CREB levels rhythmically in SCN cells and thus drive rhythmic expression of these genes. In support of this hypothesis, at E19 the circadian rhythmic expression of both genes peaked at the same time. Moreover, these peaks correspond to peak of *Nr1d1* expression rhythm, which suggests that various synchronous maternal stimuli might be involved in activation of these genes. Additionally, this finding further supports the hypothesis that *Nr1d1* expression may be driven by the maternal cues at E19 (see above). This may allow us to speculate that the initiation of clock oscillatory mechanisms is in fact related to the *Nr1d1* expression rhythm. It is unlikely that the rhythm in *Nr1d1* expression is conditional for the initiation of the fetal clock function because the clock develops autonomously even in fetuses of arrhythmic mothers without a functional clock [34]. It could, however, be responsible for proper phasing of the developing fetal clock, which is born in phase with the maternal circadian clock [15]. *Nr1d1* oscillation has already been suggested to play a role in entrainment of circadian clocks in adults [35], [36]. In accordance with this hypothesis, the maternally driven rhythmicity in the fetal SCN should be abolished in arrhythmic mothers. However, the gene expression profiles in this study resulted from population data (i.e., each time point of the profile represents fetuses of different mother) and the absence of rhythms in the fetal SCN could also be due to mutual desynchrony among the arrhythmic mothers. Therefore, the role of *Nr1d1* in maternal entrainment of the fetal SCN should be tested using other methodological approaches. In vivo recording of the bioluminescence driven by *Per1* reporter revealed daily variation in the rat fetal peripheral tissues already at E19 [37], however, such recording could not detect gene expression directly from the fetal brain and the individual nuclei.

At E21, the circadian clock appears already operational in vivo because significant rhythms in clock gene expression were detected. The functional clock likely drives expression of clock-controlled genes, namely *Avp*. In addition to CRE, the *Avp* promoter also contains an E-box sequence which binds CLOCK:BMAL1 and is thus rhythmically driven by the clock mechanism [38]. Therefore, multiple pathways originating from both maternal cues and the functional fetal clock are likely to drive *Avp* expression at E21. As a result of the mechanistic switch, the phase of *Avp* rhythm was significantly shifted and the amplitude of the rhythm increased at E21 relative to E19. The amplitude and robustness of the circadian signal further increases during the postnatal period up to P5–P10 [19], [21].

Like *c-fos* and *Avp*, P-CREB pathway may also regulate *Vip* expression [39]. *Vip* expression was rhythmic at both E19 and E21, although the rhythm was very weak compared to other evaluated rhythmic genes. Therefore, the weak circadian rhythm in *Vip* expression is present before the clock mechanism is fully functional. VIP is an important mediator among SCN cells and is responsible for increasing the robustness of their oscillations [40]. In the fetal rat central nervous system, VIP receptors are abundant already by E11 [41]. Activation of VIP receptors leads to elevation of P-CREB levels transcription of genes containing CRE in their promoter. Therefore, the above mentioned *c-fos* and *Avp* expression rhythms observed at E19 may be related to *Vip* rhythmicity via this mechanism. Additionally, VIP may play a role in synchronizing SCN neurons during the developmental period when the synaptic web is lacking. However, the amplitude of *Vip* expression rhythm is very low in the fetal SCN and it is thus not clear whether has any functional relevance.

During fetal SCN development, transcript levels changed between E19 and E21. The initially high expression levels of *Per2* and *Bmal1*, i.e., genes which were constitutively expressed at E19, declined with fetal age in correlation with the initiation of circadian regulation. In contrast, expression levels of genes that were rhythmically expressed at E19 (*Nr1d1*, *c-fos*, *Avp* and *Vip*) increased with fetal age, in correlation with clock development. These findings are in accordance with results of our previous study, in which clock gene expression in the SCN was detected by in situ hybridization [19], [21]; of the genes examined in the previous study (*Per1*, *Per2*, *Cry1*, *Bmal1* and *Clock*), *Per2* and *Bmal1* were expressed with at the highest levels at E19. The results of the present study confirm that this previous finding was indeed due to differences in transcript levels and was not related to a methodological problems related with the properties of the probes used for the in situ hybridization. Apparently, these data also suggest that with the initiation of circadian control, rhythmicity is generated via suppression of high *Per2* and *Bmal1* transcription (this study, [21]) and induction of low *Per1* and *Cry1* transcription [21].

In conclusion, our results support the hypothesis that the rat SCN circadian clock develops in vivo only after E19 and becomes functional just before birth. Before the SCN clock is operational during fetal development, various rhythms could be detected in the SCN that are likely driven by the maternal rhythmic milieu. *Nr1d1* represents the first rhythmically expressed clock gene, being rhythmic before the fetal clock begins to operate, likely due its sensitivity to the maternal cues. Therefore, based on our data, *Nr1d1* rhythmicity may represent one of the possible mechanisms for setting the fetal molecular clock with maternal cues. Nevertheless, the role of *Nr1d1* in maternal entrainment of the fetal SCN has to be proven.
